# Incidence and Risk Factors for Organ/Space Infection after Radiofrequency-Assisted Hepatectomy or Ablation of Liver Tumors in a Single Center: More than Meets the Eye

**DOI:** 10.3389/fsurg.2017.00017

**Published:** 2017-04-07

**Authors:** Ioannis Karavokyros, Stamatios Orfanos, Anastasios Angelou, Antonia Meropouli, Dimitrios Schizas, John Griniatsos, Emmanouil Pikoulis

**Affiliations:** ^1^First Department of Surgery, National and Kapodistrian University of Athens, Laikon General Hospital, Athens, Greece

**Keywords:** organ/space infection, radiofrequency, hepatectomy, bloodstream infections, surgical site infections, ablation, bacteremia

## Abstract

**Introduction:**

Surgical site infections (SSIs) and especially organ/space infection (O/SI) after resection or ablation of liver tumors are associated with increased morbidity and mortality. A secondary blood stream infection (BSI) is considered an O/SI but the exact prevalence is unknown. We aimed to investigate the incidence of O/SI and BSIs in a cohort of consecutive patients after liver resection or ablation, to seek for a possible connection between them and to search for potential risk factors.

**Materials and methods:**

We reviewed all patients who underwent hepatic resection or intraoperative liver ablation between January 2012 and December 2016 in our department. We focused on age, gender, Child–Pugh score, preoperative biliary drainage, indication for surgery, type of resection, resection or ablation of tumor, need for bilioenteric reconstruction, additional procedure to hepatectomy, blood transfusion, operative time, postoperative admission to ICU, and antibiotic chemoprophylaxis. All positive cultures from intra-abdominal fluids and blood were recorded. O/SI and BSIs were diagnosed by the criteria set by Centers for Disease Control. All variables were compared between the group with O/SI and the group without infection. BSIs were associated with these infections also.

**Results:**

Eighty-one consecutive patients with a mean age of 64 years were enrolled. Fifteen patients presented a positive culture postoperatively: intra-abdominal fluid in eight, blood cultures in six, and both blood and intra-abdominal fluid in one patient. The directly estimated incidence of O/SI amounted to 11.1%. Four blood cultures were secondary to O/SI, and the remaining two secondary to central line catheter. O/SI was diagnosed indirectly, through the BSI in an additional 4.9% of the patients, raising the incidence of SSI to 16%. Among the factors studied, only admission to the ICU was found to be statistically significant as a risk factor for the development of O/SI (*p* = 0.026).

**Conclusion:**

O/SI should be actively seeked for after liver surgery including blood cultures. Patients with affected physical status, comorbidities are in greater risk of developing O/SI.

## Introduction

Postoperative infection is a common complication after liver resection or ablation of liver tumors. It is associated with increased length of stay, morbidity, and mortality (1). Despite the recent improvements in surgical techniques and devices, hepatectomy-related surgical site infections (SSIs) account for about 1/3 of the nosocomial infections (2, 3). SSIs are classified into incisional (superficial and deep) and organ/space infection (O/SI), an important distinction due to the difference in their pathogenesis (4). O/SI occasionally leads to bacteremia and secondary blood stream infection (BSI) which in this case is also considered as SSI (4). We investigated the incidence of O/SI and BSI in a cohort of consecutive patients who underwent resection or ablation of liver tumors. We also seeked for a possible connection between them and searched for potential risk factors.

## Materials and Methods

We reviewed the medical records of all patients who underwent either radiofrequency-assisted liver resection or intraoperative radiofrequency liver ablation in our department from 2012 till 2016. Patients’ age and gender, preoperative liver function, existence of preoperative biliary stent, operative data, and postoperative need for ICU were specifically addressed. With the exception of a laparoscopic excisional biopsy, all other operations were open. All resections were RF-assisted according to the method initially described by Habib (5), and all ablations were conducted through radiofrequencies. The Cool-tip™ RF Ablation System (Medtronic Co) was employed in all operations. Drainage tube was placed in all patients. Enteral feeding was started quickly as tolerated by the patients. Antibiotic chemoprophylaxis was administered to all patients 1 h prior to the skin incision. Chemoprophylaxis scheme consisted of metronidazole combined with either cefoxitin or, in case of allergy to β-lactamic antibiotics, with ciprofloxacin. Patients with cholangitis prior to surgery were treated with piperacillin/tazobactam, and the operation was performed when all evidence of infection was eliminated and blood cultures became repeatedly negative. Patients with severe comorbidities and not ready to be extubated immediately postoperatively were transferred to ICU for a timely extubation, in compliance with the anesthesiologists’ instructions. A central venous line was inserted to all patients immediately preoperatively and removed the first postoperative day or the first post-extubation day.

Primary end-points of the study were the detection of O/SI after liver resection or ablation and the detection of BSI secondary to OSI. According to the Centers for Disease Control (CDC) definition, diagnosis of *organ/space SSI must meet the following criteria: the infection occurs within 30 days after the operative procedure if no implant is left in place or within one year if implant is in place and the infection appears to be related to the operative procedure and the infection involves any part of the anatomy (e.g., organs or spaces), other than the incision, opened or manipulated during the operative procedure. In addition, it must meet at least one of the following: purulent drainage from a drain that is placed through a stab wound into the organ/space; organisms isolated from an aseptically obtained culture of fluid or tissue in the organ/space; an abscess or other evidence of infection involving the organ/space that is found on direct examination, during reoperation, or by histopathologic or radiologic examination; or diagnosis of an organ/space SSI by a surgeon or attending physician* (6).

We discriminated primary BSI from secondary-to-SSI ones based on the CDC definition: *for a bloodstream infection to be determined secondary to another site of infection the following requirements must be met: a site-specific definition must be met; either one of the CDC/NHSN Surveillance Definitions for Specific Types of Infections, or UTI, PNEU or SSI definition AND one of the following scenarios must be met: Scenario 1: At least one organism from the blood specimen matches an organism identified from the site-specific infection that is used as an element to meet the NHSN site-specific infection criterion AND the blood specimen is collected during the secondary BSI attribution period (infection window period* + *repeat infection timeframe) OR Scenario 2: An organism identified in the blood specimen is an element that is used to meet the NHSN site-specific infection criterion, and therefore, is collected during the site-specific infection window period* (7).

Central line-associated blood stream infections (CLABSIs) were defined as *laboratory-confirmed bloodstream infection (LCBI) where central line (CL) was in place for* >*2 calendar days on the date of event, with day of device placement being Day 1, AND the line was also in place on the date of event or the day before. If a CL was in place for* >*2 calendar days and then removed, the date of event of the LCBI must be the day of discontinuation or the next day to be a CLABSI* (7).

Consequently, O/SIs were recorded by positive cultures from intra-abdominal fluids (abscess, fluids from drainage tube, etc.). Positive blood cultures with evidence or proof of O/SI were also recorded as O/SI according to the previously mentioned scenario 2 (7). We investigated a possible correlation between these infectious complications and the preoperative or perioperative parameters.

All continuous data were expressed as the mean ± SD. Chi-square test was used for statistical comparisons employing SPSS^®^ v 22 Software.

## Results

From January 2012 till December 2016, a total of 81 patients (48 males, 33 females) underwent either hepatic resection or intraoperative liver ablation in our department. As it can be seen in Table 1, the mean patient age was 64.07 ± 12.3 years old, no difference between the two sexes. Indications for liver surgery are listed in Table 2. Seventy-two patients (88.9%) underwent surgery for malignancy and 9 (11.1%) for benign tumors. Nine patients had liver ablation, 25 patients an anatomical hepatectomy, and 47 patients underwent metastasectomies or non-anatomical liver resection (Table 3). Metastasectomy was the most frequent operation (41 patients, 50.6% overall), and hepatic adenoma was the most common benign tumor (2.4% overall, 22.2% of benign tumors). No patient had a bilioenteric anastomosis prior to surgery; however, 12 patients had a biliary stent inserted preoperatively due to refractory jaundice. In these 12 patients and 7 additional ones, i.e., in 19 patients in total, bilioenteric continuity was maintained with the creation of a hepaticojejunostomy (Table 4). An additional concomitant operation, mostly a colectomy, was performed in 17 patients (Table 5). Seventy-six patients had a Child–Pugh score A, 5 patients a score B, and none a score C. No patient developed post-hepatectomy liver failure. Perioperative characteristics by occurrence of SSI are depicted in Table 6. Fifty-two patients needed blood transfusion with a mean blood transfusion of 2.1 units of packed red blood cells (range: 0–11, median: 1). With the exception of one patient suffering from lymphoma, all packed red blood cells used were not leukocyte depleted. Mean operative time for all patients was 417 ± 113 min. Sixty-seven patients received the typical chemoprophylaxis scheme. Fourteen patients were treated with piperacillin/tazobactam preoperatively. All patients had a central venous line (mostly jugular) inserted immediately preoperatively. This was removed either the first postoperative or the first post-extubation day. Nineteen patients were admitted in the ICU immediately postoperatively for a “timely extubation” due to preexisting comorbidities. ICU mean length of stay was 3.37 days (ranging 1–15 days).

**Table 1:** **Preoperative variables by occurrence of surgical site infection**.

	All patients (*n* = 81)	No postoperative infection (*n* = 66)	Postoperative infection (*n* = 15)	*p* Value
Mean age (years)	64	64	64.2	
Gender (M/F)	48/33	37/29	11/4	0.175
Child–Pugh Score (A/B)	76/5	61/5	15/0	0.651
Biliary stent (yes/no)	12/69	8/58	4/11	0.151
Malignant/benign	72/9	58/6	14/1	0.472

**Table 2:** **Indication for surgery and occurrence of positive cultures**.

Diagnosis	No.	(+)ve culture
Liver metastasis	*n*	
Colorectal adenocarcinoma	33	4
Small bowel NET	1	
Colonic NET	1	
Adrenocortical carcinoma	1	1
Malignant insulinoma	1	1^a^
Sarcoma	1	
Fallopian tube carcinoma	1	
Clear cell renal cancer	1	
Breast cancer	1	
Cholangiocarcinoma	13	5
Gallbladder cancer	4	1
Hepatocellular carcinoma	14	2
Hepatic lymphoma	1	1^a^
Liver adenoma	2	
Focal nodular hyperplasia	1	
Liver hemangioma	1	
Cholangitis	1	
Hydatidosis	1	
Liver cirrhosis	1	
Trauma	1	
Total	81	15

*^a^Central line-associated blood stream infection—blood stream infection but not surgical site infection*.

**Table 3:** **Type of liver intervention and infection occurrence**.

Type of intervention		No.	Inf
Ablation		9	2^b^
Anatomic resections		35	7
Bisegmentectomies	6		
Left lateral	7		1
Left hepatectomy	6		1
Right hepatectomy	7		2
Central hepatectomy	5		1
Extended right hepatectomy	4		2
Metastasectomies/non-anatomic resections		37^a^	7
Total		81	

*^a^1 laparoscopic excisional biopsy included*.

*^b^Central line-associated blood stream infection—blood stream infection but not surgical site infection (*Candida, Staphylococcus*)*.

**Table 4:** **Type and indication of intervention leading to bilioenteric anastomosis, occurrence of surgical site infection**.

Operation	No.	Inf	Diagnosis	No.	Inf
Extended right hepatectomy	4	2	HCC	5	1
Right hepatectomy	4	2	Cholangiocarcinoma	10	2
Central hepatectomy	5	2	Gall bladder cancer	4	4
Left hepatectomy	4				
Non-anatomic resections	2	1			
Total	19	7		19	7

**Table 5:** **Hepatic surgery combined with other operations and occurence of organ/space infections (O/SI)**.

Type of operation	No.	Diagnosis	O/SI
Right colectomy + metastasectomies	6	5 Colonic cancer + 1 colonic NET	
Left colectomy + metastasectomies	2	Colonic cancer	
Sigmoidectomy + left lateral hepatectomy	1	Colonic cancer	
Sigmoidectomy + kidney resection + metastasectomies	1	Colonic cancer + renal clear cell carcinoma	
Kidney resection + right hepatectomy	1	Renal clear cell carcinoma	
Left adrenalectomy + metastasectomies	1	Adrenocortical carcinoma	1
Small bowel resection + metastasectomies	1	Small bowel NET	
Low anterior resection + metastasectomies	3	Rectal cancer	
Low anterior resection + ablation	1	Rectal cancer	
Total	17		

**Table 6:** **Intraoperative characteristics and admission to ICU by surgical site infection occurrence**.

	All patients (*n* = 81)	No postoperative infection (*n* = 66)	Postoperative infection (*n* = 15)	*p* Value
Type of antibiotic (simple/advanced)	67/14	57/9	10/5	0.080
Resection/ablation	72/9	59/7	13/2	0.528
^a^Type of resection (anatomic/non-anatomic)	35/37	28/31	7/6	0.455
Constructed bilioenteric anastomosis (yes/no)	19/62	13/53	6/9	0.094
Additional procedure (yes/no)	17/64	16/50	1/14	0.119
Blood transfusion (yes/no)	52/29	41/25	11/4	0.307
Operative time (min)	417	411	444	0.320
ICU (yes/no)	19/62	12/54	7/8	0.026

*^a^Number of patients (*n*) = 72. Ablated patients were excluded*.

Fifteen patients (11 males, 4 females) with signs of infection presented evidence of postoperative blood or organ/space SSI. Only intra-abdominal fluid cultures were positive in eight patients while blood only cultures were positive in six patients. One patient had both intra-abdominal fluid and blood cultures positive. Table 7 shows the bacteriological data of all positive cultures. *Enterococcus* sp. was isolated in seven cultures, *Kebsiella* sp. in three, *E. coli* in two, *Pseudomonas* sp. in one, and *Serratia* sp. in one. In one blood culture, both *E. coli* and *Enterococcus* were isolated. Two bloodstream infections could not be associated to SSI, one with *Candida albicans* and the other with *Staphylococcus* coagulase (−) ve. In fact, these infections fulfilled the criteria for being characterized CLABSI and were considered as such. The remaining four BSIs were considered secondary to O/SI both by clinical and laboratory data. In conclusion, we recorded 9 patients with O/SI and 4 patients with BSI secondary to O/SI, i.e., 13 patients (16%) with SSI in total. Twelve patients had an O/SI classified as Dindo II treated only with medications. The patient with both blood culture and intra-abdominal fluid culture positive was classified as Dindo IIIA because he underwent radiological guided drainage.

**Table 7:** **Association between type of positive culture and bacteria**.

Culture type/bacteria	Blood	Intra-abdominal fluid	Blood and intra-abdominal fluid	Total
*E. coli*		1		1
*Enterococcus*	2	3	1	6
*Pseudomonas*	1			1
*Klebsiella*		3		3
*Candida*	1^a^			1
*Staphylococcus*	1^a^			1
*Serratia*		1		1
Mix culture: *Enterococcus* and *E. coli*	1			1
Total	6	8	1	15

*^a^Positive blood cultures not associated with surgical site infection but associated with central line*.

No significant difference was observed among the patients who developed organ/space or bloodstream infection regarding age, gender, Child–Pugh score, existence of preoperative biliary stent, or indication for surgery. Also, there was no significant difference in any of the perioperative parameters between the patients who developed a SSI and those who did not. In contrast, the incidence of postoperative O/SI was significantly higher in the patients needing ICU when compared to those hospitalized exclusively in the ward (36.1 vs 12.9%, *p* = 0.026).

## Discussion

Postoperative infections continue to play a significant role in morbidity and mortality of surgical patients. Especially, in major procedures like hepatectomies, infections have a high impact on length of stay, total cost and can result in postoperative death (2).

We investigated the incidence of bloodstream infection and organ/space SSI after liver surgery and searched for associated risk factors. We detected an O/SI after liver resection or ablation in 11.1% of our patients following the definition (6). This complies with the 6–10% commonly reported by others (8–10). An additional 4.9% of our patients were detected through a secondary BSI to suffer from to O/SI. In these patients, blood culture has been the sole microbiological data. In fact, two of three of our positive blood cultures had an isolated microbe matching O/SI. Consequently, the total true incidence of organ/space SSI in our cohort should increase to 16% with a difference of approximately 50%.

This difference can be easily missed for many reasons. First of all, early aggressive treatment of any infection either organ/space or bloodstream with antibiotics may cover microbiological data and obscure further the diagnosis. Second, a correlation between bloodstream infection and O/SI might not have been attempted in every case. Finally, blood microbiological data are not needed for the diagnosis of O/SI, consequently blood cultures may not be taken routinely and data may be missing. O/SIs are not always verified with microbiological data and may be diagnosed after clinical suspicion, or signs of infection followed by radiological evidence only. If these are lacking the patient may be, temporarily, discharged from the hospital, only to be readmitted later on. This is supported by the findings of Spolverato et al. where after 338 hepatectomies 14.2% of the patients were readmitted within 30 days, and 22.9% of the readmissions were due to intra-abdominal abscess (11). Along the same vein, intra-abdominal infections leading to readmission, and thus diagnosed late, represented 75% of all the intra-abdominal infections of a cohort of 712 hepatectomies (12). In both series, intra-abdominal abscess was the most common reason for readmission.

The incidence of BSI secondary to an O/SI is not clear. About 11% of 403 patients developed a post-hepatectomy BSI (13). This was considered secondary in 46% of the cases, and the source of infection was an intra-abdominal abscess in 28%. Furthermore, in 46% of the bacteremias, the onset of the infection was more than 2 weeks after hepatectomy. Clearly, in a number of patients O/SI will be diagnosed late due to the late appearance of either the primary infection or the secondary BSI.

Many risk factors and predictors have been implicated in the development of OSI. Use of silk sutures (14), postoperative bile leakage (3, 9, 15, 16), failure of the remnant liver (9), portal vein resection (1), preexisting bilioenteric anastomosis (17), and, interestingly, perioperative peritoneal lavage (18) have all been associated with increased rates of OSIs. In contrast, use of broad spectrum antibiotics in chemoprophylaxis (17) as cefuroxime alone confers no protection (19), employment of minimally invasive techniques (20), and avoidance of postoperative bile leak through the air leak test (21) seem to confer a prophylactic effect. Preexisting chronic liver disease and cirrhosis (22) and ERCP and stenting of the common bile duct (17) seem to have no effect on either SSI or OSI.

Perioperative blood loss, transfusion of blood or blood products have been reported also to have no effect on the development of SSI (23, 24) although others disagree (15). Our data indicate that blood transfusion has no effect on the development of SSI in liver surgery. However, we cannot draw any conclusion on the effect of the type of transfused blood, as described in colorectal surgery (25), and suggested by Okabayashi et al. (15) because only one of our patients received leukocyte-depleted blood.

A similar debate seems to exist regarding concomitant bowel surgery: it has been reported that it has no effect on the incidence rates of OSI (1, 24, 26) while according to others it leads to increased infection rates (27). Operating on the right colon presents a different infective morbidity compared to colectomies of the hindgut (28) but evidence regarding the combination of liver and colorectal surgery is missing. Our results imply that the addition of another procedure to hepatectomy has no effect on O/SI. In our cohort, neither midgut nor hindgut resections were followed by an infective complication. The only significant risk factor we were able to point out was postoperative admission to the ICU. This association may be well explained by the overall status of the patients. As most of the operative data were similar for those developing SSI and those who did not, postoperative need for ICU reflects the comorbidities and the bad performance status. Conducting a major operation to these fragile patients rendered them susceptible to infection, increasing morbidity.

Our study has the disadvantage of being a small retrospective study comprising of only 81 consecutive cases of hepatectomy or intraoperative liver ablation. Perhaps, a greater number of patients would allow us to exhibit the significance of additional risk factors as reported in the literature. However, these are well described and probably we would not add anything new to the existing knowledge. What we aimed at was a focused investigation in O/SIs after hepatectomy, with emphasis in secondary bloodstream infections. We believe that true O/SIs are more frequent than reported because many cases are not identified or are identified late and not included in the published reports. Alternatively, they may be classified as infections of other type. Of course, larger studies are needed to confirm our observation and perhaps to identify additional risk factors. In this manner, we will be able to provide special care to the vulnerable patients being at higher risk.

## Ethics Statement

This study was specifically reviewed and approved by the ethics committee of our institutional review board with written informed consent from all subjects. All subjects gave written informed consent in accordance with the Declaration of Helsinki.

## Author Contributions

IK, SO, and AA designed the study. SO, AA, AM, and DS collected and analyzed the patients’ data. SO and IK wrote the paper. JG critically revised the draft. EP supervised the manuscript.

## Conflict of Interest Statement

The authors declare that the research was conducted in the absence of any commercial or financial relationships that could be construed as a potential conflict of interest. The handling Editor declared a shared affiliation, though no other collaboration, with the authors and states that the process nevertheless met the standards of a fair and objective review.

## Abbreviations

SSI, surgical site infection; O/SI, organ/space infection; BSI, blood stream infection; CLABSI, central line-associated blood stream infection; CDC, Centers for Disease Control.
